# Wireless Sensing and Networking for the Internet of Things

**DOI:** 10.3390/s23031461

**Published:** 2023-01-28

**Authors:** Zihuai Lin, Wei Xiang

**Affiliations:** 1 School of Electrical & Information Engineering, University of Sydney, Camperdown, NSW 2006, Australia; 2 School of Engineering and Mathematical Sciences, La Trobe University, Melbourne, VIC 3086, Australia

In recent years, we have witnessed the exponential proliferation of the Internet of Things (IoT)-based networks of physical devices, vehicles, and appliances, as well as other items embedded with electronics, software, sensors, actuators, and connectivity, which enable these objects to connect and exchange data. Facilitating the introduction of highly efficient IoT, wireless sensing, and network technologies will reduce the need for traditional processes that must currently be manually carried out, thus freeing up the precious resources of a dwindling workforce, and informing more meaningful and necessarily human-centered work.

This Special Issue aims to collate innovative developments in areas relating to IoT, wireless sensing, and networking. The eighteen papers published in this Special Issue cover software-defined network (SDN)-based IoT networks, artificial intelligence (AI) for IoT, industrial IoT, smart sensors, energy efficiency optimization for IoT and wireless sensor networks, IoT applications for agriculture, smart cities, healthcare, localization, and environment monitoring.

In [1], an IoT network with intercept access points (IAPs), SDN nodes, and non-SDN nodes was developed for the purpose of lawful interception. Different from traditional networks with centralized management, this paper optimized the deployment of IAPs in hybrid software-defined networks containing both SDN and non-SDN nodes. This work presented an enhanced equal-cost multi-path shortest-path algorithm for IAP deployment and three SDN interception models in accordance. In addition, the authors proposed the use of a restriction minimal vertex cover algorithm (RMVCA) in hybrid SDN nodes to consider the geographic importance of all intercepted targets and the global cost of operator operations and maintenance. By applying a variety of SDN interception algorithms based on the RMVCA to actual network topologies, the authors were able to significantly optimize the deployment efficiency of IAPs and improve the intercept link coverage in hybrid SDN nodes, as well as reasonably deploy the best intercept access point and intercept the whole hybrid SDN with the fewest SDN nodes, thereby aiding in the introduction of lawful interception.

The second paper [2] developed anomaly detection methods by utilizing machine learning to safeguard an IoT system. The authors provided a thorough analysis of prior work in creating machine-learning-based anomaly detection methods for safeguarding IoT systems. Additionally, they claimed that blockchain-based systems used for anomaly detection are capable of jointly building efficient machine learning models for anomaly detection.

The authors of [3] outlined a comprehensive self-testing method that used energy-efficient learning modules and nanoscale electromagnetic (EM) sensing devices to identify security concerns and malicious attacks at the front-end sensors. The development of a built-in threat detection method employing intelligent EM sensors dispersed on the power lines was proven to facilitate the efficient use of energy while detecting unusual data activity without compromising performance. Energy-constrained wireless devices may also be able to have an on-chip detection system to quickly foresee hostile attacks on the front lines due to the minimal energy and space usage.

Ref. [4] introduced a D2D multi-criteria learning technique for secured IoT networks to enhance data exchange without adding extra fees or data diversions for mobile sensors. Additionally, machine learning was shown to lower the risks of compromise in the presence of anonymous devices and increase the reliability of the IoT-enabled communication system. Broad simulation-based experiments were also used to evaluate and assess the proposed work, showing significantly better performance for realistic network topologies in terms of packet delivery ratio, packet disruptions, data delays, energy consumption, and computing complexity.

The authors of [5] demonstrated how machine learning can improve the functionality of biosensors without biological receptors. The performance of these biosensors was enhanced by machine learning, which effectively substitutes modeling for the bioreceptor to increase specificity. Since their introduction, simple regression models have been commonly used in biosensor-related fields to determine analyte compositions based on the biosensor’s signal strength. Traditionally, bioreceptors offer good sensitivity and specificity to the biosensor. However, a growing number of biosensors without bioreceptors have been created for a variety of purposes. The usage of ML for imaging, E-nose and E-tongue, and surface-enhanced Raman spectroscopy (SERS) biosensors was discussed in this study. It is also particularly noteworthy that several artificial neural network (ANN) methods paired with principal component analysis (PCA), support vector machine (SVM), and other algorithms performed remarkably in a variety of tasks.

The authors of [6] stressed the exigency of using a virtual testbed dubbed IoTactileSim to implement, investigate, and manage QoS provisioning in tactile industrial IoT (IIoT) services. The study demonstrated that tactile IIoT enables the real-time control and manipulation of remote industrial environments via a human operator. The authors also showed that a communication network with ultra-low latency, ultra-high reliability, availability, and security is required by TIoT application cases. Furthermore, it has become more difficult to research and enhance the quality of services (QoSs) for tactile IIoT applications due to the absence of the tactile IIoT testbed. IoTactileSim uses the robotic simulator CoppeliaSim and network emulator Mininet to carry out real-time haptic teleoperations in both virtual and actual surroundings. This allows the real-time monitoring of network impairments, operators, and teleoperator data flow, as well as various implemented technology parametric values.

In [7], a novel feature fusion-based approach to scene text detection was created. Rather than solely relying on feature extraction from SENet, this technique incorporated MPANet’s features to make up the difference. By using the suggested fusion technique, the text detection model could achieve better detection performance than the baseline network. In addition, the model was post-processed with a progressive expansion technique to provide rapid and precise text detection. This method was shown to be important for in studying natural scene text detection technology that is oriented toward actual application scenarios because it aims to improve experimental results without introducing end-to-end networks with too many parameters, and it ultimately achieves high accuracy and fast text detection.

The energy-efficient design of IoT is a very challenging topic. As mentioned in [8], although IoT technologies and paradigms such as edge computing have enormous potential for the digital transition towards sustainability, they do not yet contribute to the IoT industry’s sustainable development. Due to its use of scarce raw materials and its energy consumption in manufacturing, operation, and recycling processes, this industry has a substantial carbon footprint. To address these challenges, the green IoT (G-IoT) paradigm was developed as a study field to lower this carbon footprint; nevertheless, its sustainable vision directly clashes with the arrival of edge artificial intelligence (edge AI), which mandates the use of additional energy. The authors of [8] addressed this issue by investigating various factors that influence the design and development of edge AI G-IoT systems. In addition, their study provided an Industry 5.0 use case that highlights the various principles that were discussed. In particular, the proposed scenario involved an Industry 5.0 smart workshop that aims to improve operator safety and operation tracking, employing a mist computing architecture built of IoT nodes with AI capabilities.

For the energy harvesting of IoT in paper [9], a fast and accurate numerical method was given to determine the RF–DC power conversion efficiency (PCE) of energy harvesting circuits in the case of power-carrying signals with multiple tones and periodic envelopes. In recent years, extensive research has been conducted on this kind of signal. For low-to-medium input power levels, their use was shown to produce a potentially higher PCE than the usual sine wave signal. Because of this, the authors wanted to devise a fast and accurate two-frequency harmonic balance method (2F-HB) because a fast PCE calculation could speed up the process of optimizing the converter circuit by a lot. A comparison study was conducted to show how well the 2F-HB works when it comes to computing. The results of [9] show that the 2F-HB performs much better than widely used methods such as the transient analysis (TA) method, the harmonic balance method (HB), and the multidimensional harmonic balance method (MHB). This method also proved to be more effective than Keysight ADS, a commercial non-linear circuit simulator that uses both HB and MHB. The proposed method could also be easily added to commercially available non-linear circuit simulation software, such as Keysight ADS and Ansys HFSS, as used by many people.

Unmanned aerial vehicles (UAVs) represent one of the new types of devices that use 5G and 6G networks. One possible way of supporting advanced services for UAVs, such as video monitoring, is to use the recently standardized millimeter-wave (mmWave) frequency band for new radio (NR) technology. However, buildings may cause frequent outages if they block the paths between NR base stations (BSs) and UAVs. In [10], the authors used the tools of integral geometry to describe the connectivity properties of UAVs in terrestrial urban deployments of mm-wave NR systems. The main metric of interest is the likelihood of UAV line-of-sight (LoS) blockage. Unlike other studies, the proposed approach made it possible to obtain a close approximation of the likelihood of line-of-sight blockage as a function of city and network deployment parameters.

In another review [11], early-stage coverage path planning (CPP) methods were presented in the robotics field. The objective of CPP algorithms is to reduce the overall coverage path and execution time. Significant research has been conducted in the field of robotics, particularly in the areas of multi-unmanned unmanned aerial vehicle (UAV) collaboration and energy efficiency in CPP challenges. In addition, this paper also addressed multi-UAV CPP techniques and focused on CPP algorithms that conserve energy.

In [12], the authors investigated a method used to mitigate the user’s body shadowing effect on the RSSI to improve localization accuracy. They also examined the effect of the user’s body on the RSSI. The idea of a landmark was then used to develop an angle estimate method. An inertial measurement unit (IMU)-aided decision tree-based motion mode classifier was used to accurately identify different landmarks. A compensation strategy was then proposed to fix the RSSI. The closest neighbor method was used to estimate the unknown location. The results show that the suggested system can greatly increase localization accuracy. After adjusting for the body effect, a median localization accuracy of 1.46 m was attained, compared to 2.68 m before the compensation using the traditional K-nearest neighbor approach. Additionally, when comparing the suggested system’s performance to that of the two other relevant works, it clearly surpassed the competition. By using a weighted K-nearest neighbor approach, the median accuracy was further increased to 0.74 m.

Direction-of-arrival (DOA) estimation is integral in array signal processing, and the estimating signal parameter via rotational invariance techniques (ESPRIT) algorithm is one of the typical super-resolution algorithms used for finding directions in an electromagnetic vector sensor (EMVS) array. However, existing ESPRIT algorithms treat the output of the EMVS array as a “long vector”, which leads to a loss of signal orthogonality. Ref. [13] proposed a geometric algebra-based ESPRIT algorithm (GA-ESPRIT) to estimate 2D-DOA with double parallel uniform linear arrays. The approach integrated GA with ESPRIT to describe multidimensional signals holistically. Direction angles were determined by different GA matrix operations to retain correlations among EMVS components. Experimental results show that GA-ESPRIT is robust to model mistakes and requires less time and memory.

The ‘15-min city’ concept offers new perspectives on livability and urban health in post-pandemic cities. Smart city network technologies can offer personalized pathways to respond to contextualized difficulties through data mining and processing to better enhance urban decision-making processes. The authors of [14] argued that digital twins, IoT, and 6G can benefit from the ‘15-min city’ concept. The data collected by these devices and analyzed by machine learning reveal urban fabric patterns. Unpacking these dimensions to support the ‘15-min city’ notion can illuminate new ways of redefining agendas to better respond to economic and societal requirements and line with environmental commitments, including UN Sustainable Development Goal 11 and the New Urban Agenda. This study argued that these new connectivities should be examined so that relevant protocols can be created and urban agendas can be recalibrated to prepare for impending technology breakthroughs, offering new avenues for urban regeneration and resilience crafting.

Environment monitoring is one of the commonly used IoT applications. Ref. [15] proposed a low-latency LoRaWAN system for environmental monitoring in factories at major accident risk (FMARs). Low-power wearable devices for sensing dangerous inflammable gases in industrial plants are meant to reduce hazards and accidents. Detected data must be provided immediately and reliably to a remote server to trigger preventive steps and then optimize the functioning of a machine. In these scenarios, the LoRaWAN system is the best connectivity technology due to off-the-shelf hardware and software. The authors examined LoRaWAN’s latency and reliability restrictions and proposed a strategy to overcome them. The suggested solution also used downlink control packets to synchronize ED transmissions (DCPs). These experiments validated the proposed technique for the FMAR scenario.

For low-cost IoT precision agriculture applications such as greenhouse sensing and actuation, the authors of [16] created a LoRaWAN-based wireless sensor network with low power consumption. All of the research’s subsystems were entirely constructed using only commercially available components and freely available or open-source software components and libraries. This entire system was established to demonstrate the possibility of creating a modular system using low-cost commercially available components for sensing purposes. The data generated by the experiments were compiled and kept in a database maintained by a cloud-based virtual computer. Using a graphical user interface, the user had the ability to observe the data in real time. In a series of experiments conducted with two types of natural soil, loamy sand and silty loam, the overall system’s dependability was demonstrated. The system’s performance in terms of soil characteristics was then compared to that of a Sentek reference sensor. Temperature readings indicate good agreement within the rated accuracy of the implemented sensors, whereas readings from the inexpensive volumetric water content (VWC) sensor revealed variable sensitivity. The authors made several conclusions using a unique approach to maximize the parameters of the non-linear fitting equation connecting the inexpensive VWC sensor’s analog voltage output with the standard VWC.

The authors of [17] integrated LPWAN technology to an existing proximate soil sensor device by building an attachment hardware system (AHS) and accomplishing technical upgrades for low-cost, low-power, wide-coverage, and real-time soil monitoring in fields. The testing results demonstrate that, after upgrading, the sensor device can run for several years with only a battery power supply, and that the effective wireless communication coverage is nearly 1 km in a typical suburban farming context. As a result, the gadget not only keeps the sensor device’s original mature sensing technology, but also displays ultra-low power consumption and long-distance transmission. The proposed method also serves as a model for extending LPWAN technology to a broader spectrum of inventoried sensor devices for technical advancements.

The final paper [18] of this Special Issue focused on digital twins for cattle care. The authors established cutting-edge artificial-intelligence-powered digital twins of cattle status in this research (AI). The project was based on an IoT farm system that can record and monitor the health of livestock from a distance. The sensor data obtained from the farm IoT system was used to create a digital twin model of cattle based on deep learning (DL). It was shown that the real-time monitoring of the physiological cycle of cattle is possible, and by applying this model, the next physiological cycle of cattle can be predicted. An enormous amount of data to confirm the accuracy of the digital twins model acted as the foundation of this effort. The loss error of training for this digital twin model, predicting the future behavioral state of cattle, was approximately 0.580, and the loss error of doing so after optimization was approximately 5.197. This work’s digital twins model could be used to predict the cattle’s future time budget.
